# The carcinogenicity of opium consumption: a systematic review and meta-analysis

**DOI:** 10.1007/s10654-023-00969-7

**Published:** 2023-02-11

**Authors:** Adalberto M. Filho, Michelle C. Turner, Saman Warnakulasuriya, David B. Richardson, Bayan Hosseini, Farin Kamangar, Akram Pourshams, Vikash Sewram, Deirdre Cronin-Fenton, Arash Etemadi, Deborah C. Glass, Afarin Rahimi-Movaghar, Mahdi Sheikh, Reza Malekzadeh, Mary K. Schubauer-Berigan

**Affiliations:** 1 International Agency for Research On Cancer, Lyon, France; 2 Barcelona Institute for Global Health (ISGlobal), Barcelona, Spain; 3 Universitat Pompeu Fabra (UPF), Barcelona, Spain; 4 CIBER Epidemiología Y Salud Pública (CIBERESP), Madrid, Spain; 5 King’s College London, London, UK; 6 University of California, Irvine, CA USA; 7 Morgan State University, Baltimore, USA; 8 Digestive Oncology Research Center, Digestive Diseases Research Institute, Tehran University of Medical Sciences, Tehran, Iran; 9 Department of Global Health, African Cancer Institute, Stellenbosch University, Stellenbosch, South Africa; 10 Department of Clinical Epidemiology, Department of Clinical Medicine, Aarhus University, Aarhus, Denmark; 11 Division of Cancer Epidemiology and Genetics, National Cancer Institute, Maryland, USA; 12 School of Public Health and Preventive Medicine, Monash University, Melbourne, Australia; 13 Iranian National Center for Addiction Studies, Tehran University of Medical Sciences, Tehran, Iran

**Keywords:** Opium, Meta-analysis, Cancer, Cohort study, Case–control study

## Abstract

The carcinogenicity of opium consumption was recently evaluated by a Working Group convened by the International Agency for Research on Cancer (IARC). We supplement the recent IARC evaluation by conducting an extended systematic review as well as a quantitative meta-analytic assessment of the role of opium consumption and risk for selected cancers, evaluating in detail various aspects of study quality on meta-analytic findings. We searched the published literature to identify all relevant studies on opium consumption and risk of selected cancers in humans through 31 October, 2022. Meta-relative risks (mRRs) and associated 95% confidence intervals (CIs) were estimated using random-effects models for studies of cancer of the urinary bladder, larynx, lung, oesophagus, pancreas, and stomach. Heterogeneity among studies was assessed using the *I*^*2*^ statistic. We assessed study quality and conducted sensitivity analyses to evaluate the impact of potential reverse causation, protopathic bias, selection bias, information bias, and confounding. In total, 2 prospective cohort studies and 33 case–control studies were included. The overall pooled mRR estimated for ‘*ever or regular*’ versus ‘*never*’ use of opium ranged from 1.50 (95% CI 1.13–1.99, *I*^*2*^ = 0%, 6 studies) for oesophageal cancer to 7.97 (95% CI 4.79–13.3, *I*^*2*^ = 62%, 7 studies) for laryngeal cancer. Analyses of cumulative opium exposure suggested greater risk of cancer associated with higher opium consumption. Findings were robust in sensitivity analyses excluding studies prone to potential methodological sources of biases and confounding. Findings support an adverse association between opium consumption and cancers of the urinary bladder, larynx, lung, oesophagus, pancreas and stomach.

**Supplementary Information:**

The online version contains supplementary material available at 10.1007/s10654-023-00969-7.

## Introduction

Opium is consumed as an illicit recreational narcotic drug, or for medicinal purposes, in more than 50 countries worldwide, with the highest prevalence observed in Western Asia [1]. Opium is typically either ingested or smoked. Opium is the dried latex from the unripe seed-pods of the opium poppy plant. Commonly consumed forms include raw (or crude) opium, opium dross (tarry residues formed after smoking raw opium), and minimally refined opium or opium sap (boiled opium dross with or without added raw opium). Opium contains several addictive alkaloids [2] and (when burned) pyrolysates and may also be contaminated with lead, chromium, and arsenic [3, 4]. Since the 1970s, epidemiological studies conducted in regions of high opium consumption have suggested a positive association between frequent use of opium and cancer risk [5–7]. Recent findings from the large-scale Golestan cohort study (GCS) [8]—a cohort of 50,000 individuals followed for more than a decade—have also highlighted adverse associations of opium consumption and cancer risk at several sites.

In 2020, the carcinogenicity of opium was evaluated by a Working Group convened by the International Agency for Research on Cancer (IARC), and opium consumption was classified as “carcinogenic to humans” (Group 1) with *sufficient evidence* of carcinogenicity in humans for cancers of the urinary bladder, larynx, and lung, and *limited evidence* for cancers of the oesophagus, stomach, pancreas, and pharynx [2]. Although positive associations were observed, chance, bias, and confounding could not be ruled out by the Working Group for cancer sites where *limited evidence* was observed [2].

Here, we aimed to supplement the recent qualitative IARC cancer hazard evaluation by conducting an extended systematic review and a quantitative meta-analytic assessment of the role of opium consumption and risk for selected cancers, including cancers of the urinary bladder, larynx, lung, oesophagus, stomach, and pancreas [9]. This analysis therefore represents the most comprehensive systematic review and meta-analysis to date [10–14]. We specifically considered in detail exposure assessment quality, and the impact of various potential methodological sources of bias and confounding on meta-analytic findings according to a registered protocol.

## Methods

### Literature search and inclusion criteria

The protocol for this systematic review and meta-analysis was registered in Prospero, number CRD42021236030. We conducted extended searches of the published literature to identify all relevant analytical epidemiological studies, comprising cohort and case–control studies on opium consumption and cancer risk in humans. Epidemiological studies that examined the association between opium consumption and cancers of the urinary bladder, larynx, lung, oesophagus, stomach, and pancreas were included because the IARC *Monographs* evaluation concluded that there was either *sufficient* or *limited* evidence in epidemiological studies, and there were at least 3 studies identified for each cancer site in the peer-reviewed literature. Pancreatic cancer was included in the analysis a posteriori of the Prospero protocol publication, because extended literature searches here identified additional publications since the meeting of the IARC Working Group (held during 11–20 September 2020), thereby meeting the criteria for inclusion. No language restrictions were applied. Although there are a few studies on opium use and the risk of other cancers, including cancers of the colon and rectum, brain, liver, lip, oral cavity, and pharynx, due to the small number of available studies for each of these sites, they did not meet the inclusion criteria for this review.

AMF and MCT conducted an extended search of the published literature without time restriction of start date, through 31 October 2022, by searching the following bibliographic databases, namely PubMed, EMBASE and Web of Science. The following MeSH terms were used in: PUBMED: (opium[tw] OR (8006-60-4[m]) AND (neoplasm* OR carcinogen* OR malignan* OR tumor* OR tumour* OR cancer*), and in EMBASE, Mtree-Terms-expanded included: opium:ti,ab,kw AND (neoplasm* OR carcinogen* OR malignan*OR tumor* OR tumour* OR cancer). A detailed description of the online search strategy is available in the Health Assessment Workspace Collaborative (HAWC) software [15] at the following website: https://hawcproject.iarc.fr/assessment/675/. All titles and abstracts were screened by AMF and MCT, followed by screening of the manuscript’s full text where appropriate. All studies that met the eligibility criteria were included in critical appraisal and meta-analysis. There were no exclusions due to study quality or other methodological factors. In some instances, manuscripts reported only information on the prevalence of opium consumption among study participants, but the study was included if measures of association could be calculated (see “Data extraction” below). In the case of multiple publications from the same study, only the most recent or relevant was included. In one included study (Khoo et al. [6]), the exposure reported was opium and/or heroin; there was no information on the proportion of heroin users.

In this systematic review and meta-analysis, we aimed to identify studies according to the following PECO statement, namely: Population (men and women), Exposure (opium consumption by either smoking or oral ingestion), Comparators (members of the same source population with no or minimal opium consumption), and Outcome (incident or fatal cancer of the urinary bladder, larynx, lung, oesophagus, stomach, or pancreas), considering also the time component T (aspects of follow-up, to minimize the potential for protopathic bias or reverse causation). Studies that examined opium consumption prior to cancer occurrence or mortality were identified, with studies of cancer incidence preferred to those of mortality when both were available in the same study population.

### Exposure evaluation

Exposure assessment in the included studies was performed either prospectively or retrospectively in relation to cancer outcomes. Prospective exposure assessment occurred where the pattern of opium consumption was captured at study enrolment, and subsequent cancer incidence was examined over follow-up time. In such studies, the risk of cancer was assessed among exposed vs unexposed participants, as well as by intensity of exposure measured at baseline (categories of duration or cumulative use, often in the local unit of nokhod-years [16]). Studies with retrospective exposure assessment were mainly case–control studies of the general population or of patients diagnosed with cancer for whom previous exposure to opium consumption had been evaluated and compared with that of persons without the cancer of interest. This meta-analysis includes only studies that assessed exposure to minimally processed forms of opium (e.g., raw opium, opium dross, minimally refined opium). Populations exposed only to other opiates (e.g., morphine, codeine), semi-synthetic opioids (e.g., heroin, oxycontin), or synthetic opioids (e.g. fentanyl) were not included here. In all primary studies, opium consumption by current or former users was captured through either self- or proxy- completed questionnaires, interviews, or extracted from patient medical records. Consumption of opium was categorized in most studies as ‘ever-user’ or ‘regular user’ versus ‘never user’ of opium. For some studies, duration (years) of opium use and/or cumulative opium consumption were available. Definitions of these categories varied across the studies. For example, in the GCS and several studies that used the GCSQ questionnaire, ‘*regular*’ use of opium was defined as use at least once per week for at least 6 months. Infrequent users who did not meet this definition were likely included in the ‘*never*’ use category.

### Data extraction

Information on study characteristics, design, and results was extracted by IARC *Monographs* Working Group members, or by AMF and MCT for newly identified studies published after the completion of the *Monographs* meeting in September 2020, and verified for completeness and accuracy [2]. Information on a range of study features was extracted including: (a) opium consumption measures (form of opium, route of consumption, cumulative exposure); (b) incident cancer outcome (the only mortality studies identified were of the GCS, which was superseded by an incidence study); (c) socio-demographic characteristics of the study participants, when available; (d) information on potential confounders included in the analyses (with a primary focus on age, sex, tobacco smoking); (e) measure of association (e.g., odds ratio, hazard ratio) and associated 95% confidence intervals (CIs).

Studies in which opium exposure was assessed as "drug addiction" are assumed here to be either smoking or ingestion. Exposure referred to as “snuffing” in Bakhshaee et al. [17], was assumed to be smoking. ORs were estimated either by IARC *Monographs* Working Group members [18, 19], or (for Khoo and colleagues [6]) estimated elsewhere [13]. Two other included studies [20, 21] provided only the total number of cases and controls and numbers of exposed, so the point estimate and 95% CI were estimated by the authors here. In two studies [22, 23], categories of opium use were combined by the authors here. Nourbakhsh et al. [24] and Tootoonchi et al. [25] were excluded from tabulation in the Monograph [2] because they were considered minimally informative, due to a lack of information on the analysis, population characteristics, and/or exposure to opium, but are included here in our meta-analysis for completeness for the overall analysis.

Quality assessment of each included publication was performed independently by two reviewers (AMF and MCT) using five primary quality assessment criteria defined by the Working Group in relation to studies of opium consumption and cancer risk [2]. Any discrepancies between the two reviewers were resolved by a third reviewer (MSB). The study quality assessment criteria pertained to five factors, including the potential for (1) reverse causation (whether the diagnosis of a cancer type of interest causes a change in opium consumption), (2) protopathic bias (whether opium is consumed in response to symptoms of the cancer type of interest that is still undiagnosed at the time of data collection), (3) selection bias (whether enrolment of study participants is related to both opium consumption and the cancer outcome), (4) information bias (primarily considering the potential for recall bias), and (5) confounding [the extent to which major confounders (including age, sex, and tobacco smoking) were adequately considered, and whether residual confounding of findings was likely to be substantial] for each cancer outcome. Studies were classified by cancer sites into categories of either major, medium, or low concern for each of these five factors, and the potential magnitude and direction of bias were considered (see Appendix). Sensitivity analysis was conducted by excluding studies with major concern for a given factor (see Appendix) and also by including only case–control studies (more details provided in “Statistical models”).

Studies adjusted for tobacco smoking, age and sex were considered to be of higher quality. Smoking-adjusted estimates were preferred in this analysis when available; however, the assessment of the importance of tobacco smoking as a potential confounder varied depending on the strength of the association between tobacco smoking and the cancer site (e.g., for cancers of lung and larynx, tobacco smoking was considered a strong potential confounder).

### Statistical analysis

Meta relative-risk (mRR) estimates and associated 95% CIs were calculated using random-effects models to integrate generic inverse variance data. The main parameter estimated is a categorical mRR for ‘*ever* or *regular*’ compared with ‘*never*’ consumption of opium. Given the paucity of available cohort studies, summary mRR estimates were computed using results from all case–control and cohort studies combined (using the rare disease assumption for estimates based on case–control studies [26]). We used generic inverse-variance data and pooled the estimates using random effect models. In brief, we transformed the log values for the odds ratio and relative risk. The standard error was calculated using the 95% confidence interval bounds. To estimate the variance between studies, we employed the Sidik–Jonkman adjustment, which is used in confidence interval estimation when the sample size is small [27]. The adjustment is based on an improvement of the variance parameters, which is performed by combining the variance of the sample size and the sample variance [28].

A series of sensitivity analyses was conducted by excluding studies identified during the quality assessment as having potential major bias or confounding, for example, due to failure to sufficiently account for reverse causation or protopathic bias, or lack of adjustment for important confounders (including age, sex, and tobacco smoking). Heterogeneity among the studies was evaluated using I^2^ and τ^2^ (Tau-square) values (we considered values of *I*^*2*^ = 0–25% as representing low heterogeneity, 26–50% moderate heterogeneity and 50–100% high heterogeneity [29]). To investigate potential publication bias we used funnel plots and Begg’s statistic tests.

Although most of the included studies characterised exposure as a dichotomous variable (see above), some studies provided more detailed information on the amount of opium consumed, duration of consumption, and/or cumulative consumption. For these studies, meta-analysis was conducted to further explore associations between the categories of cumulative opium consumption and cancer risk. Cumulative exposure information was available for some studies of cancers of the urinary bladder [8, 30, 31, 43], larynx [8, 32], lung [8, 33, 34], pancreas [8, 35, 36] and stomach [8, 37, 55] (never versus ≤ median and > median use). Estimates of association between ≤  median and > median of cumulative opium exposure compared to never use for various cancers in the GCS [8], and for bladder cancer in the IROPICAN study [38] were obtained through personal communication with the study authors (November 2021, and April 2022, respectively). The models included adjustment for age, sex, and tobacco smoking.

Overall, results fully adjusted for confounding, including tobacco smoking, were used where available. For cancer sites with available results in three or more studies of never-users of tobacco, results were calculated where possible. Statistical analysis was performed using the library “meta” (version. 5.0) [39] in R software v4.1 [40].

## Results

### Study identification

**Fig. 1:**
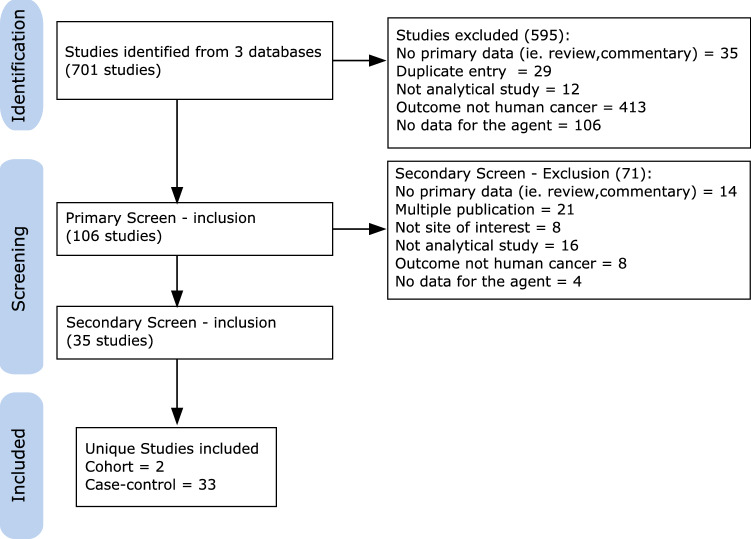
Flowchart showing details of the inclusion end exclusion criteria here

Figure 1 presents details of the inclusion and exclusion criteria to select articles for the systematic review and meta-analysis. In total, 701 studies were identified by the comprehensive literature search after removing duplicates. A total of 106 studies were retained following the first screening of titles and abstracts and the exclusion of studies that were not relevant. Following a second screen of the full text of the manuscripts, 35 studies were included (one publication [41] included two studies). These 35 studies then underwent quality assessment and data abstraction.

### Characteristics of studies

Table 1 describes the studies included in the systematic review of opium consumption and cancer, by potential confounding variables considered and route of exposure. All studies were conducted in Asia, almost exclusively in Iran, and were published between the years 1969 and 2022. There were 2 prospective cohort studies and 33 case–control studies. There were a total of six new case–control studies identified following the *Monographs* meeting [30, 35, 38, 42–44]. A total of 15 studies examined the association between opium consumption and bladder cancer (1 cohort and 14 case–control), 7 for laryngeal cancer (1 cohort and 6 case–control), 6 for oesophageal cancer (1 cohort and 4 case–control, one publication [41] describes two separate studies, with different sets of cases and controls), 5 for stomach cancer (2 cohort and 3 case–control), 5 for lung cancer (1 cohort and 4 case–control), and 4 for pancreatic cancer (1 cohort and 3 case–control). The studies included approximately 8400 cancer cases, of whom 2947 reported opium consumption (bladder cancer, n = 1091; oesophageal cancer, n = 295; laryngeal cancer, n = 545; lung cancer, n = 536; pancreatic cancer, n = 231; and stomach cancer, n = 249). The main routes of opium consumption were through smoking and/or ingestion. Most studies provided estimates for both routes combined, while 62% (n = 22) of studies presented risk estimates for consumption by smoking and 40% (n = 17) for consumption by ingestion separately. Most of the studies (77%, n = 27) provided estimates adjusted for age, sex, and tobacco smoking. Most case–control studies used hospital-based controls (33 studies) and one study used neighbourhood controls. As noted above, opium consumption was primarily assessed through interview-based questionnaires. One cohort study (GCS) used a questionnaire at baseline, and exposure estimates from the questionnaire were confirmed by measuring opium consumption metabolites in urine for a sample (n = 150 participants) of the study cohort [45].

**Table 1:** Description of the studies included in the systematic review and meta-analysis of opium consumption and cancer, by type of confounding variables adjusted and route of exposure

	Region	n exposed cases	n total participants	Enrollment period	Study design	Variables controlled^a^			Route of exposure^b^	
						Age	Sex	Smoking	Smoking	Ingestion
*Bladder*										
Abdolahinia et al.2021 [30]	Kerman, Iran	74	300	2020	case–control	X	X	X		
Akbari et al. 2015 [31]	Shiraz,Iran	43	594	2012–2013	case–control	X	X	X		
Aliasgari et al. 2004 [18]	Tehran, Iran	13	160	1997–2000	case–control		X	X		
Aliramaji et al. 2015 [19]	Babol, Iran	58	350	2001–2012	case–control	X	X	X		
Ghadimi et al. 2015 [46]	Kurdistan, Iran	16	304	2012–2014	case–control	X	X	X	X	
Hadji et al. 2022 [38]	Multicentric, Iran	303	4149	2017–2020	case–control	X	X	X	X	X
Hosseini et al. 2010 [47]	Tehran, Iran	60	358	2004–2008	case–control	X	X	X	X	X
Ketabchi et al. 2005 [48]	Kerman, Iran	80	300		case–control	X	X	X		
Lotfi et al. 2016 [49]	Yazd, Iran	52	400	2009–2013	case–control	X	X			
Nourbakhsh et al. 2006 [24]	Tehran, Iran	41	510	1990–2000	case–control	X	X	X		
Rashidian et al. 2021 [43]	Kerman, Iran	200	900	2013–2015	case–control	X	X	X	X	X
Sadeghi et al. 1979 [22]	Shiraz, Iran	45	198	1969–1976	case–control	X	X	X		
Shakhssalim et al. 2010 [50]	Tehran, Khorasan, Khoozestan, Isfahan and East Azarbayjan, Iran	67^c^	1384	2006	case–control	X	X	X		
Sheikh et al. 2020 [8]	Golestan, Iran	23	50,034	2004–2008	cohort	X	X	X	X	X
Tootoonchi et al. 2000 [25]	Esphahan, Iran	16	284		case–control	X	X	X		
*Larynx*										
Alizadeh et al. 2020 [32]	Kerman, Iran	88	420	2014–2017	case–control	X	X	X	X	
Bakhshaee et al. 2017 [17]	Mashhad, Iran		85	2008–2010	case–control	X	X	X	X	
Berjis et al. 2018 [51]	Isfahan, Iran	101	360	2015	case–control			X		
Khoo et al. 1981 [6]	Hong Kong	27	246	1970–1977	case–control	X	X			
Mohebbi et al. 2021 [52]	Iran (10 provinces)	231	3392	2016–2019	case–control	X	X	X	X	X
Mousavi et al. 2003 [53]	Kerman, Iran	75	410	1996–2002	case–control	X	X	X		
Sheikh et al. 2020 [8]	Golestan, Iran	23	50,034	2004–2008	cohort	X	X	X	X	X
*Lung*										
MacLennan et al. 1977 [5]	Singapore	63	533	1972–1973	case–control	X	X		X	
Masjedi et al. 2013 [33]	Tehran, Iran	33	726	2002–2005	case–control	X	X	X	X	X
Naghibzadeh-Tahami et al. 2020 [34]	Kerman, Iran	83	420	2014–2017	case–control	X	X	X	X	X
Rashidian et al. (2022) [42]	Multicentric, Iran	300	4104	2017–2020	case–control	X	X	X	X	X
Sheikh et al. 2020 [8]	Golestan, Iran	57	50,034	2004–2008	cohort	X	X	X	X	X
*Oesophagus*										
Bakhshaee et al. 2017 [17]	Mashhad, Iran	*-*	123	2008–2010	case–control	X			X	
Hakami et al. 2014 [20]	Golestan and Fars, Iran	13	120		case–control	X	X			
Pournaghi et al. 2019 [23]	North Khorasan, Iran	54	283	2013–2015	case–control	X	X		X	X
Shakeri et al. 2012 [41]	Golestan, Iran	45	390	2002–2003	case–control	X	X	X	X	X
Shakeri et al. 2012* [7]	Golestan, Iran	90	871	2004–2007	case–control	X	X	X	X	X
Sheikh et al. 2020 [8]	Golestan, Iran	93	50,034	2004–2008	cohort	X	X	X	X	X
Pancreas										
Naghibzadeh-Tahami et al. 2021[35]	Kerman, Iran	77	528	2016–2018	case–control	X	X	X	X	X
Sanat et al. 2021 [44]	Tehran, Iran	75	996	2012–2018	case–control	X	X	X		
Shakeri et al. 2016 [36]	Tehran, Iran	57	685	2011–2015	case–control	X	X	X		
Sheikh et al. 2020 [8]	Golestan, Iran	22	50,034	2004–2008	cohort	X	X	X	X	X
*Stomach*										
Karajibani et al. 2014** [54]	Zahedan, Iran	12	92	2011–2012	case–control	X	X			
Naghibzadeh-Tahami et al. 2014 [37]	Kerman, Iran	34	426	2010–2012	case–control	X	X	X		
Sadjadi et al. 2014 [21]	Ardabil, Iran	4	928		cohort	X	X	X		
Shakeri et al. 2013 [55]	Golestan, Iran	109	922	2004–2011	case–control	X	X	X		
Sheikh et al. 2020 [8]	Golestan, Iran	90	50,034	2004–2008	cohort	X	X	X	X	X

^*^Shakeri et al. 2012/Nasrollahzadeh et al. 2008

^**^values obtained from proportions (23.9% and 2.2%)

^a^Adjustment or matching

^b^when X is not present = both routes combined

^c^ = refers to the n for “history of opium consumption”

Several case–control (n = at least 9) studies also used the GCS questionnaire (GCSQ) [7, 31, 32, 34, 35, 37, 41, 52, 55]. A similarly detailed questionnaire was also used in the IROPICAN study [38, 42, 43, 52]. Other case–control studies ascertained exposure information from telephone calls, face-to-face interviews, patient records or public demographic information and usually characterised exposure as ever/never opium use [5, 17, 19, 22–24, 33, 46, 47, 49–51, 53]. The GCS [8] provided risk estimates for all the cancer sites investigated here, had high quality exposure information (collected using the GCSQ), as nokhod-years, and distinguished between ingested and smoked opium. For cancer of the oesophagus, two case–control studies had higher quality exposure information [7, 41]. Three of the four lung cancer case–control studies [33, 34, 42], two of the six laryngeal cancer case–control studies [32, 52], and one of the three pancreatic cancer case–control studies [35, 36, 44] also had higher quality exposure information with cumulative exposure estimates. Some bladder cancer case–control studies relied on patient records and did not define a minimum exposure for ‘*regular*’ user (hence the estimate could be interpreted as for an ‘*ever*’ user). For bladder cancer, three case–control studies [30, 31, 38] and for stomach cancer, three of the four case–control studies [37, 55] had a measure of intensity of exposure. Exposure quality considerations for most of these studies are extensively described in the published monograph [2].

For each study included in this review, study quality appraisal and assessment of the potential for bias and confounding are provided in the Appendix. The majority of studies were assessed as having either medium or low concern regarding reverse causation or protopathic bias, with the exception of studies of laryngeal cancer and lung cancer where there were major concerns for some studies for these items, as well for confounding. There were major concerns regarding selection and information bias (specifically, exposure misclassification) among studies of several cancer sites.

### Risk estimates and heterogeneity

**Fig. 2:**
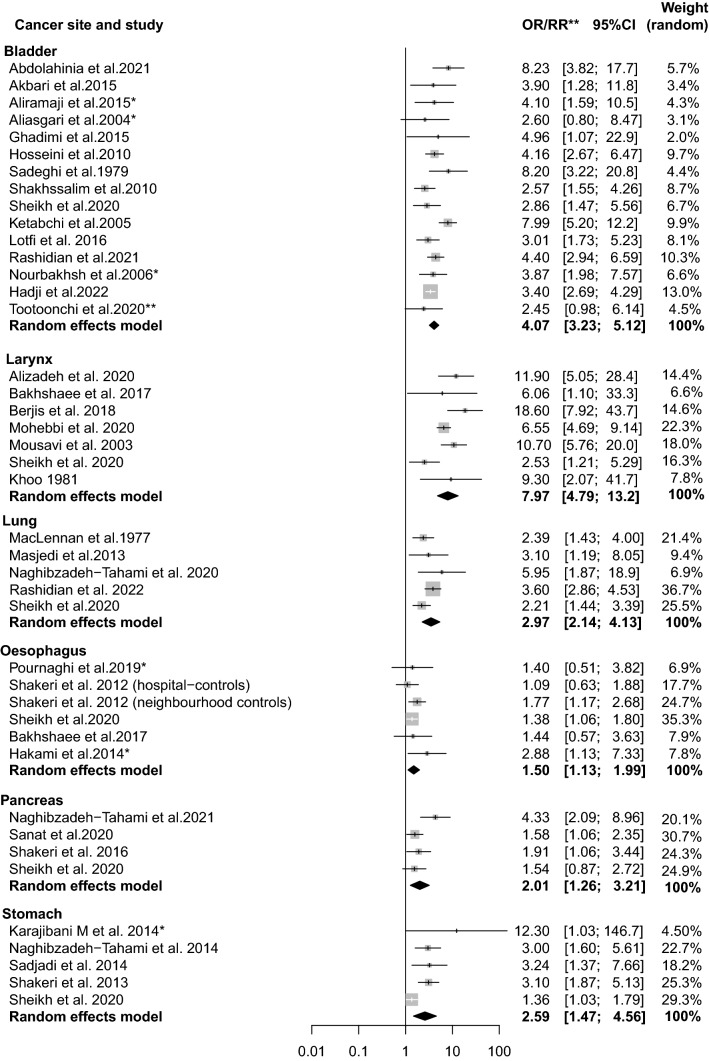
Forest plot for opium consumption and cancer risk, including all studies. (*Note*: OR/RR = odds-ratio/rate-ratio, *Estimated by the authors, ** both tobacco-adjusted or crude estimates.)

**Fig. 3:**
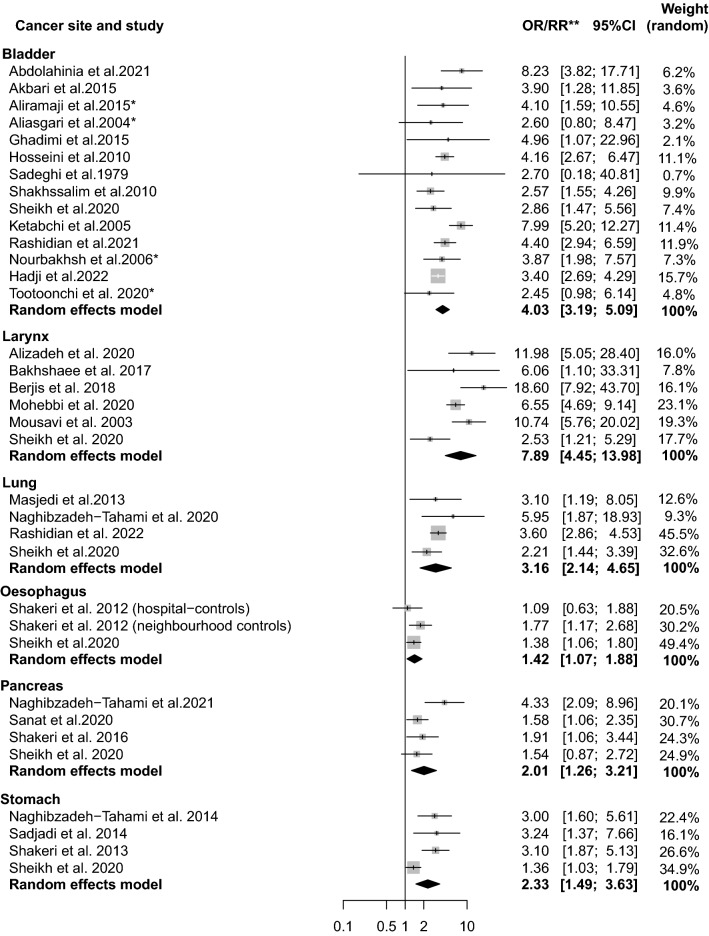
Forest plot for opium consumption and cancer risk, including only studies that adjusted for tobacco smoking (*Note*: OR/RR = odds-ratio/rate-ratio)

Figure 2 shows a pooled forest plot mRRs (n = 41 study estimates) for opium consumption and cancer from all 35 included studies. Overall, the pooled estimates ranged from 1.50 (95% CI 1.13–1.99), *I*^2^ = 0% for cancer of the oesophagus to 7.97 (95% CI 4.79–13.2), *I*^2^ = 62% for cancer of larynx. Pooled mRR estimates for ‘*ever* or *regular*’ opium consumption compared to ‘*never*’ consumption among only studies adjusting for tobacco smoking (n = 35 study estimates) were similar (Fig. 3). The tobacco-adjusted pooled estimates ranged from 1.42 (95% CI 1.07, 1.88), *I*^2^ = 2% for cancer of oesophagus to 7.89 (95% CI 4.45, 13.98), *I*^2^ = 68% for cancer of larynx.

Table 2 shows results of the meta-analysis and sensitivity analyses for the risk of cancer and opium consumption. Overall, there were high levels of heterogeneity observed in most analyses for cancers of larynx, pancreas, and stomach. In an analysis including all studies, heterogeneity was low in the analysis of cancer of the oesophagus (0%), but higher amounts of heterogeneity were observed in analyses of other cancer sites. In the analysis including only case–control studies, observed heterogeneity was reduced for cancers of the lung (0%, n = 3), larynx (42%, n = 5), and stomach (0%, n = 2), but not for bladder and pancreatic cancer. In results of sensitivity analysis that excluded studies of Toutounchi et al. (2000) [25] and Nourbakhsh et al. (2006) [24] that were not considered by the IARC Working Group evaluation (above), findings for bladder cancer were generally similar overall (mRR = 4.19 (95%CI 3.26–5.38), *I*^*2*^ = 49%, and *tau*^*2*^ 0.09) and among studies adjusting for tobacco smoking (mRR = 4.15 (95%CI 3.22–5.36), *I*^*2*^ = 45%, and *﻿tau*^*2*^ 0.08).

**Table 2:** Results of the meta-analysis and sensitivity analyses for opium consumption and cancer risk

Analyses^a^	N	mRR	95%CI	*I*^*2*^	tau^2^	References included
*Bladder*						
All studies included	15	4.07	3.23–5.12	43%	0.09	[8, 18, 19, 22, 24, 25, 30, 31, 38, 43, 46–50]
Adjustment for tobacco smoking	14	4.03	3.19–5.09	39%	0.08	[8, 18, 19, 22, 24, 25, 30, 31, 38, 43, 46–48, 48]
Only case–control studies	13	4.14	3.24–5.29	41%	0.08	[18, 19, 22, 24, 25, 30, 31, 38, 43, 46–48, 48]
No major concerns for Confounding	14	4.03	3.19–5.09	39%	0.08	[8, 18, 19, 22, 24, 25, 30, 31, 38, 43, 46–48, 48]
No major concerns for Reverse causation	14	4.03	3.19–5.09	39%	0.08	[8, 18, 19, 22, 24, 25, 30, 31, 38, 43, 46–48, 48]
No major concerns for Selection bias	5	3.40	2.70–4.30	0%	0.02	[8, 31, 38, 43, 50]
No major concerns for Information bias	5	3.69	3.01–4.41	0%	0.01	[8, 31, 38, 43, 47]
No major concerns for Protopathic bias	14	4.03	3.19–5.09	39%	0.08	[8, 18, 19, 22, 24, 25, 30, 31, 38, 43, 46–48, 48]
Never tobacco user	4	3.81	2.70–5.37	0%	0.00	[8, 19, 22, 38]
*Oesophagus*						
All studies included	6	1.50	1.13–1.99	0%	0,04	[7, 8, 17, 20, 23, 41]
Adjustment for tobacco smoking	3	1.42	1.07–1.88	2%	0,02	[7, 8, 41]
Only case–control studies	2	1.44	0.89–2.30	48%	0,06	[23, 41]
No major concerns for Confounding	3	1.42	1.07–1.88	2%	0,02	[7, 8, 41]
No major concerns for Reverse causation	3	1.42	1.07–1.88	2%	0,02	[7, 8, 41]
No major concerns for Selection bias	2	1.50	1.15–1.96	0%	0,01	[8, 41]
No major concerns for Protopathic bias	3	1.42	1.07–1.88	2%	0,02	[7, 8, 41]
*Larynx*						
All studies included	7	7.97	4.79–13.2	62%	0.27	[6, 8, 17, 32, 51–53]
Adjustment for tobacco smoking	6	7.89	4.45–13.98	68%	0.34	[8, 17, 32, 51–53]
Only case–control studies	5	9.64	6.27–14.8	42%	0.10	[17, 32, 51–53]
No major concerns for Confounding	5	6.69	3.80–11.8	63%	0.26	[8, 17, 32, 52, 53]
No major concerns for Reverse causation	3	5.78	2.49–13.4	75%	0.44	[8, 32, 52]
No major concerns for Selection bias	3	5.78	2.49–13.4	75%	0.44	[8, 32, 52]
No major concerns for Information bias	4	6.76	1.21–5.29	72%	0.36	[8, 32, 52, 53]
No major concerns for Protopathic bias	3	5.78	2.49–13.4	75%	0.44	[8, 32, 52]
*Lung*						
All studies included	5	2.97	2.14–4.13	35%	0.06	[5, 8, 33, 34, 42]
Adjustment for tobacco smoking	4	3.16	2.14–4.65	39%	0.07	[8, 33, 34, 42]
Only case–control studies	3	3.69	2.61–5.20	0%	0.67	[33, 34, 42]
No major concerns for Confounding	4	3.16	2.14–4.65	39%	0.07	[8, 33, 34, 42]
No major concerns for Reverse causation	3	3.20	1.94–5.26	59%	0.12	[8, 34, 42]
No major concerns for Selection bias	3	3.20	1.94–5.26	59%	0.12	[8, 34, 42]
No major concerns for Information bias	3	3.20	1.94–5.26	59%	0.12	[8, 34, 42]
No major concerns for Protopathic bias	4	3.16	2.14–4.65	39%	0.07	[8, 33, 34, 42]
*Pancreas*						
All studies included	4	2.01	1.26–3.21	52%	0.14	[8, 35, 36, 44]
Adjustment for tobacco smoking	4	2.01	1.26–3.21	52%	0.14	[8, 35, 36, 44]
Only case–control studies	3	2.22	1.24–4.00	65%	0.19	[35, 36, 44]
No major concerns for Confounding	4	2.01	1.26–3.21	52%	0.14	[8, 35, 36, 44]
No major concerns for Reverse causation	4	2.01	1.26–3.21	52%	0.14	[8, 35, 36, 44]
No major concerns for Selection bias	2	2.51	0.95–6.60	79%	0.38	[8, 35]
No major concerns for Information bias	3	2.26	1.23–4.14	60%	0.19	[8, 35, 36]
No major concerns for Protopathic bias	4	2.01	1.26–3.21	52%	0.14	[8, 35, 36, 44]
*Stomach*						
All studies included	5	2.59	1.47–4.56	73%	0.26	[8, 21, 37, 55, 54]
Adjustment for tobacco smoking	4	2.33	1.49–3.63	76%	0.13	[8, 21, 37, 55]
Only case–control studies	2	3.06	2.07–4.53	0%	0.00	[37, 55]
No major concerns for Confounding	4	2.33	1.49–3.63	76%	0.13	[8, 21, 37, 55]
No major concerns for Reverse causation	4	2.33	1.49–3.63	76%	0.13	[8, 21, 37, 55]
No major concerns for Selection bias	4	2.33	1.49–3.63	76%	0.13	[8, 21, 37, 55]
No major concerns for Information bias	3	2.13	1.21–3.75	74%	0.16	[8, 21, 37]
No major concerns for Protopathic bias	4	2.33	1.49–3.63	76%	0.13	[8, 21, 37, 55]

^a^When < 2 studies presented low or medium concern for bias or confounding, estimates were not calculated here. Note the IARC *Monographs* Working Group placed greater emphasis on tobacco-adjusted findings in their evaluation. A more detailed examination of various potential methodological sources of bias (reverse causation, protopathic bias, selection bias, information bias) and confounding (considering major potential confounders and the extent of concern for residual confounding) was additionally examined here, with findings presented based on studies that had tobacco-adjusted findings

Many of the studies were evaluated as having major concern for selection and information biases, ranging from 20% (stomach cancer) to 67% of studies (oesophageal cancer), and 17% (bladder cancer) to 83% (oesophageal cancer), respectively. The exclusion of studies identified with major concern for reverse causation, protopathic bias, selection bias, information bias, and confounding (primarily related to control for age, sex, tobacco smoking) did not result in important changes in the magnitude of the mRR in analysis for most cancer types and did not decrease the level of heterogeneity among the studies. Visual inspection of the funnel plot and Begg’s test for asymmetry did not suggest any important publication bias (p = 0.07). For bladder cancer (the only site with 3 or more results available among never smokers), the OR for ‘*regular* or *ever*’ (compared with ‘*never*’) use of opium was 3.81 (95% CI 2.70–5.37),* I*^2^ = 0% among never-users of tobacco (Table 2).

We also conducted a meta-analysis according to categories of cumulative exposure to opium consumption, comparing a lower-exposed group (≤ median) and higher-exposed group (> median) to never-users of opium where possible (Table 3). Except for lung cancer, results suggest a monotonic increase in the mRRs across the low-to-high categories of opium consumption (e.g., bladder cancer mRRs for the low and high categories of cumulative exposure were 3.70 and 4.87, respectively), but 95% CIs were wide on these estimates.

**Table 3:** Pooled mRR summary and 95% CIs of opium consumption and cancer risk, according to categories of cumulative exposure

	N	mRR	95%CI	*I*^*2*^	tau^2^	References included
*Bladder*	4					[8, 30, 31, 43]
Low category of cumulative exposure		3.70	2.16–6.32	9%	0.10	
High category of cumulative exposure		4.87	3.08–7.68	43%	0.11	
*Larynx cancer*	2					[8, 32]
Low category of cumulative exposure		4.24	0.92–19.61	84%	1.00	
High category of cumulative exposure		5.79	1.78–18.85	75%	0.53	
*Lung*	3					[8, 33, 34]
Low category of cumulative exposure		3.72	1.40–9.94	78%	0.54	
High category of cumulative exposure		3.63	1.90–6.90	27%	0.11	
*Pancreas*	3					[8, 35, 36]
Low category of cumulative exposure		2.04	0.93–4.48	51%	0.28	
High category of cumulative exposure		2.43	1.17–5.05	58%	0.26	
*Stomach*	3					[8, 37, 55]
Low category of cumulative exposure		2.28	1.02–5.06	61%	0.33	
High category of cumulative exposure		3.33	1.10–10.02	88%	0.78	

Rashidian et al. (2022) [42] did not provide cumulative exposure by low (≤ median) or high (> median) categories of cumulative exposure compared to never users

Shakeri et al. 2013 (median = 50 hookah-years)

Shakeri et al. 2016 (median = 34 nokhod-year)

Masjedi et al. 2013 (median = 36.5 nokhod/day)

Naghibzadeh et al. 2020 (median = 87.5 g-years)

Abdolahinia et al. 2021 (1800 g-year in lifetime)

Sheikh et al. 2020 (median = 21 nokhod-years)

Rashidian et al. 2022 (not provided)

## Discussion

Our study provides the most up-to-date systematic review and quantitative assessment of the association between opium consumption and cancers of the bladder, lung, larynx, pancreas, stomach and oesophagus, enhancing the evidence presented in the *IARC Monographs* volume 126 [2]. This meta-analysis found a positive association between ‘*ever* or *regular*’ (vs. ‘*never*’) exposure to opium consumption and cancers of the bladder, lung, larynx, stomach, oesophagus, and pancreas. Across the cancer sites, opium ‘*ever* or *regular’* consumers had a 1.5–eightfold increased risk of cancer when compared with those ‘*never’* consuming opium, with the strongest associations observed for cancers of the larynx and urinary bladder. These cancer sites, together with lung, may represent organs of greatest initial or elimination exposure to opium or its metabolites [2], although definitive evidence is lacking.

Substantial levels of heterogeneity were found for studies of cancer of the larynx and stomach (which were noted in the *IARC Monograph* to have a lower proportion of studies with good-quality exposure assessment); nevertheless, the summary mRRs remained similar in magnitude across a variety of sensitivity analyses. High levels of heterogeneity may reduce the reliability of the pooled estimates of mRR. Tobacco smoking has been previously classified as having *sufficient* evidence for its carcinogenicity in humans for cancers of the bladder, lung, larynx, stomach, and oesophagus [56, 57]. However, it is unlikely that residual confounding from tobacco smoking alone explains the high heterogeneity found for cancers of the stomach and larynx. Residual confounding from tobacco smoking might be expected to result in higher heterogeneity for cancer of the lung and larynx, given its strong association with these cancers. Moreover, cancers of the stomach and larynx represent a wide range of histological subtypes, mostly adenocarcinomas or squamous cell carcinomas, which have different behaviours and probable aetiology. Lack of adjustment for other known risk factors in some studies may have contributed to the heterogeneity, for example, hot beverages for oesophageal cancer [58], and *Helicobacter pylori* [59] for stomach cancer, although the degree of association between these factors and opium consumption is unknown. Consumption of alcoholic beverages was considered to present a minimal threat of confounding, given the low prevalence of use in the studied populations [53].

Differences in exposure assessment quality among the studies, which were substantial [2], could have contributed to the observed study heterogeneity. One major potential source of heterogeneity was the variable definition of ‘*regular* or *ever*’ opium consumption in the included studies. Furthermore, because of limitations in exposure assessment methodology, numbers for the intensity and duration of opium consumption and the cumulative exposure distribution were seldom available in the reviewed studies. Overall, our systematic review shows a wide range of exposure evaluation approaches among the studies; most studies provided only dichotomous classification, while few others provided cumulative exposure evaluation, as either quartiles or tertiles of exposure. Five studies provided median values of cumulative exposure, although they used different metrics (“hookah-years”, nokhod/day-years or g/years). Using the information on median consumption provided by four studies on bladder cancer, three studies on lung, stomach, and pancreas cancers, and two on larynx cancer, we estimated exposure–response relations for cancer risk across cumulative exposure categories (never-users vs lower-than-median and higher-than-median consumption). Our pooled estimates suggested a positive exposure–response relation for most cancer sites. Evidence from the GCS for several cancer sites [8] provides the most precise and relevant information for exposure–response associations between metrics of opium use (duration, cumulative use) and risk of lung cancer, and to a lesser extent for stomach cancer.

The GCSQ, also used in several case–control studies, provided high-quality exposure information including cumulative exposure estimates. Sheikh et al. [8] used the cumulative exposure estimates to show an exposure–response trend which is an indicator of causality. However, the cohort studies collected the opium exposure estimates at baseline, and were not updated over follow-up time; therefore, exposures may have changed.

One important source of heterogeneity in the mRRs not explored here was the geographic variability across Iran in the prevalence and amount of opium consumption. For example, opium consumption rates are lower in the Golestan Province [60] than in some other regions of Iran where epidemiologic studies have been conducted [2]. Furthermore, cumulative exposure based on nokhods or grams consumed may not be accurate, and there is evidence that people may have underestimated opium use in nokhods and overestimated use in grams of opium consumed and that this varies by geographical region [61].

This quantitative assessment aims to supplement the qualitative conclusions made by the *IARC Monographs* volume 126 Working Group evaluation. The study quality appraisal here includes qualitative aspects considered by the Working Group in the evaluation of the carcinogenicity of opium consumption, with clear and transparent indicators [2]. In our approach, we classified the domains in five categories related to reverse causation, protopathic bias, selection bias, information bias, and confounding. Findings were robust in a range of sensitivity analyses excluding studies with major concern regarding bias or confounding.

Exclusion of studies that did not adjust for tobacco smoking generally had little impact on findings as did restriction of included studies for bladder cancer to those of never-users of tobacco only. Otherwise, as expected, the magnitude of the summary findings, in general, tended to decrease with exclusion of studies with major concern. For example, the strength of the association of opium consumption and cancer of the larynx decreased from 7.97 (95% CI 4.79–13.2) to 5.78 (95% CI 2.49–13.4) with exclusion of studies with major concern for either reverse causation or protopathic bias, of particular concern here as disease symptoms may be alleviated by opium use (i.e., cough). For selection bias, however, studies with major concern may be biased downwards, in the event that selection is related with opium use, for example in a case–control study using unhealthy hospital controls. We found here, however, in most instances, that estimates decreased with exclusion of studies with major concern for selection bias.

The members of the IARC Monographs Working Group calculated three ORs (two for bladder cancer [18, 19], and one for cancer of the larynx [6]). A sensitivity analysis that excluded these studies, however, did not show any significant variation in the pooled estimates or of heterogeneity compared with the main findings (data not shown).

We used a comprehensive and transparent study quality appraisal that independently evaluated major concerns for potential sources of bias and confounding instead of the GRADE system [62], which has been developed for clinical decision-making and is of questionable relevance for synthesis of observational studies of environmental exposures [63, 64]. Additionally, the studies included here were conducted almost exclusively in Iran, which could pose challenges to the generalizability of the findings and may not be representative of use in other countries. Further research to better characterize opium consumption and potential health and cancer risks more globally is warranted*.*

In one of the few studies performed outside of Iran [6], opium exposure was poorly defined, and an unknown number of heroin users were likely included as part of the opium addicted group. Although opium exposure was poorly defined, is it difficult to speculate how exposure misclassification may impact cases and controls (other cancers) and study findings here.

All studies included in our meta-analysis evaluated cancer risk in relation to exposure to minimally processed forms of illicit opium (e.g., sukteh, teriak). Such “street” opium may contain contaminants and adulterants that are considered integral parts of the complex mixture to which opium users are exposed. Raw opium is formed from more than 25 types of alkaloids representing the major components (10–12%), including phenanthrenes (e.g., morphine, codeine and thebaine) and benzylisoquinolines (e.g., papaverine and noscapine), and non-alkaloids (e.g., sugars, proteins, fats and water among others) [65, 66]. Raw opium is often adulterated and/or contaminated with heavy metals, including some well-known carcinogens (e.g., lead, arsenic, chromium, cadmium [67]). A recent study from the GCS found that blood lead levels were linked to opium usage, for both oral and smoking forms [3].

We observed the strongest associations for smoking-related cancers (bladder, larynx, and lung). This is consistent with findings that opium users are exposed to many of the same carcinogens as tobacco users [68]. *IARC Monographs* vol. 126 concluded that there was *strong* mechanistic evidence of genotoxicity for opium dross (sukhteh) and opium pyrolysates (solid residues of combusted opium), in both human peripheral blood mononuclear cells and Chinese hamster ovary cells [69]. Despite scant research, one study in humans exposed to opium also suggests a genotoxic effect of frequent p53 mutations in opium users [70]. More recently, a mutational signature was identified for opium among cases of oesophageal cancer in humans [71]. The carcinogenicity of opium in animal models is still unclear, as no high-quality in vivo experimental carcinogenicity studies have yet been conducted.

Despite the limitations inherent to systematic reviews and meta-analyses, we carefully conducted our review with transparent methods and inclusion and exclusion criteria defined before the data collection. Our sensitivity analysis explored several important sources of bias and heterogeneity, with minimal effects on the results. The results consider estimates adjusted for the main potential confounders (age, sex, tobacco smoking) and stratified by cancer types. Due to limitations in the reporting of most of the studies, we were not able to provide summary risk estimates by quantitative categories of cumulative opium consumption. One limitation of most studies was that the referent category might have included opium users (current and/or past) of low frequency or intensity, underestimating the risk. We found little evidence of publication bias; one notable feature of some of the studies is that opium consumption was not the main exposure being evaluated in primary studies, which may have reduced this potential bias. We also conducted an appraisal of the most important aspects of study quality, and documented a lack of cumulative exposure information, which might be further explored in future studies of opium consumption and cancer risk. Although other meta-analyses have been recently published on opium consumption and cancer risk [10–14], our systematic review is the most up to date, and has carefully considered a range of methodological sources of bias in findings, according to domains of study quality assessment defined by the *IARC Monographs* volume 126 Working Group.

Our meta-analysis findings provide further support of the *IARC Monographs* volume 126 Working Group, which classified opium consumption as *carcinogenic to humans* (Group 1), with *sufficient* evidence for a causal association for cancers of the bladder, lung and larynx, and *limited* evidence for a causal association for cancers of pancreas, stomach, and oesophagus [2, 72]. We observed, among studies that adjusted for tobacco smoking, a substantially higher risk of cancers of the bladder and larynx among ever compared with never opium consumers, and somewhat lower elevations in risk for cancers of the lung, stomach, pancreas, and oesophagus. We also observed evidence of positive exposure–response patterns for most cancer sites. The quantitative estimates provided here lend support for developing policies for cancer prevention and control, particularly increasing the awareness of associated hazards of use among opium consumers in affected regions.

### Electronic supplementary material

Below is the link to the electronic supplementary material.

Supplementary file1 (DOCX 92 KB)

## Appendix

List 1. Quality appraisal in cohort and case–control studies of opium exposure and cancer. (Three main categories: major concern, medium concern, low concern)

1. Reverse causation (defined outcome of interest causes a change in the exposure of interest)
2. Protopathic bias (if a person uses opium in response to a symptom of an outcome of interest that is, at the time of exposure, still undiagnosed; and, if those with symptoms have a higher probability of the outcome).
3. Selection bias.
4. Information bias, including recall bias.
5. Confounding.
