# Supplementary material for: The carcinogenicity of opium consumption: a systematic review and meta-analysis
Source: Eur J Epidemiol. 2023 Feb 11;38(4):373–89. doi: 10.1007/s10654-023-00969-7 (PMC10082119; doi:10.1007/s10654-023-00969-7)
Supplement: Supplementary file 1 — Supplementary file1 (DOCX 92 KB) [file 10654_2023_969_MOESM1_ESM.docx]

**Supplement**

**The carcinogenicity of opium consumption: a systematic review and meta-analysis**

Adalberto Miranda-Filho^1^, Michelle C. Turner^2-4^, Saman Warnakulasuriya^5^, David B. Richardson^1,6^, Bayan Hosseini^1^, Farin Kamangar^7^, Akram Pourshams^8^, Vikash Sewram^9^, Deirdre Cronin-Fenton^12^, Arash Etemadi^10^, Deborah C. Glass^11^, Afarin Rahimi-Movaghar^13^, Mahdi Sheikh^1^, Reza Malekzadeh^8^, Mary K. Schubauer-Berigan^1^

Table of Contents

[Supplementary Figure 1. Funnel plot for the metanalysis on opium consumption and cancer. Dashed lines represent the region with 95% of studies fits in the absence of heterogeneity 2](#_Toc121137734)

[Supplementary Table 2. Quality appraisal in cohort and case–control studies of opium exposure and cancer. (Three main categories: major concern, medium concern, low concern). 3](#_Toc121137735)

# Supplementary Figure 1. Funnel plot for the metanalysis on opium consumption and cancer. Dashed lines represent the region with 95% of studies fits in the absence of heterogeneity

#

# Supplementary Table 2. Quality appraisal in cohort and case–control studies of opium exposure and cancer. (Three main categories: major concern, medium concern, low concern).

Legend: White (Low concern), Light blue (Medium concern), Dark blue (Major concern)

1. Reverse causation:

Low concern: Prospective cohort with outcome of interest free participants enrolled and opium use assessed at baseline; Case-control study with reliable assessment and analysis of opium use before diagnosis; Case-control study excluding users who recently started using opium in analysis

Medium concern: Case-control study with unclear or unreliable assessment of timing of opium use pre- or post-diagnosis

Major concern: Case-control study with unclear or unreliable assessment of timing of opium use pre- or post-diagnosis and disease symptoms alleviated by opium use (ie. cough)

2. Protopathic bias:

Low concern: Prospective cohort with symptomatic individuals excluded at baseline or excluding outcome events in early follow-up period; Case-control study with reliable assessment and analysis of opium use in a pre-symptomatic period before onset of disease symptoms; Case-control study excluding users who started using opium during symptomatic period; Case-control study adjusting for relevant confounders (ie. cigarette smoking)

Medium concern: Case-control study including users who started using opium during a symptomatic period or unclear

Major concern: Case-control study including users who started using opium during a symptomatic period or unclear and disease symptoms alleviated by opium use (ie. cough)

3. Selection bias:

Low concern: Prospective cohort with participant selection unrelated with opium or outcome of interest; Case-control study with control selection unrelated with opium use (i.e. non-hospital controls with exposure prevalence near general population or healthy hospital controls)

Major concern: Case-control study with selection potentially related with opium use (i.e. unhealthy controls or unclear)

4. Information bias

Low concern: Prospective cohort with opium use assessed at baseline captured before disease diagnosis; Case-control study with opium use based on records prior to diagnosis of outcome; Case-control study with opium use based on self-report with reasonable attempt to minimize information/recall bias

Major concern: Case-control study with opium use based on self-report

5. Confounding

Low concern: Prospective cohort with analyses adjusted or restricted according to major confounders of interest (age, sex, tobacco smoking), major confounders measured well and residual confounding of low concern; Case-control study with analyses adjusted or restricted according to major confounders of interest (age, sex, tobacco smoking), major confounders measured well and residual confounding of low concern;

Medium concern: Case-control study with analyses adjusted or restricted according to major confounders of interest though some residual confounding is expected

Major concern: Case-control study with analyses unadjusted or not restricted according to major confounders of interest, no matching performed, major confounders measured inadequately, residual confounding is of concern
